# Cardioembolic Stroke and ST-Segment Elevation Myocardial Infarction (STEMI) Due to Left Atrial Myxoma in an Elderly Pilgrim: A Case Report

**DOI:** 10.7759/cureus.99433

**Published:** 2025-12-17

**Authors:** Hanan Almalki, Fayez G Aldarsouni, Bahaaedin Saleh, Abdalla Malda, Emad Alamoudi

**Affiliations:** 1 Department of Critical Care Medicine, Makkah Ajyad Emergency Hospital, Makkah, SAU; 2 Department of Vascular and Endovascular Surgery, Besançon Regional University Hospital, Besançon, FRA; 3 Department of Cardiology, Dr. Samir Abbas Hospital, Jeddah, SAU; 4 Department of Trauma Surgery, King Saud Medical City, Riyadh, SAU

**Keywords:** cardiac neoplasm, cardioembolic stroke, echocardiography, hajj and umrah, left atrial myxoma, mass gathering medicine, pulseless electrical activity arrest, transthoracic and transesophageal echocardiography

## Abstract

Left atrial myxomas are the most common primary cardiac tumors, but may present deceptively. A 77-year-old woman with type 2 diabetes collapsed during *Tawaf* (circumambulation of the Kaaba in Makkah) with pulseless electrical activity arrest. Return of spontaneous circulation was achieved, and subsequent evaluation revealed a large left atrial myxoma complicated by inferior ST-segment elevation myocardial infarction (STEMI) and multiple posterior circulation infarcts. Echocardiography demonstrated a mobile septal mass prolapsing into the left ventricle, while brain imaging confirmed bilateral cerebellar and pontine strokes. The patient was stabilized, started on antithrombotic therapy after hemorrhage was excluded, and later transferred for surgical excision. This case illustrates the unusual convergence of cardiac arrest, myocardial infarction, and embolic stroke from a single atrial myxoma. It emphasizes the importance of early echocardiography in avoiding misdiagnosis and inappropriate treatment. In the setting of Hajj and Umrah (Islamic pilgrimages to Makkah), where elderly pilgrims with diverse comorbidities gather, structural cardiac lesions, though uncommon, should be considered in the differential diagnosis of sudden collapse.

## Introduction

Left atrial myxoma is the most common primary cardiac tumor, representing 30-50% of benign cardiac tumors [1], but its diagnosis remains uncommon and often delayed, with an incidence of only 0.05% [2]. Despite their benign histology, these tumors carry malignant potential through embolization or intracardiac obstruction. Neurological complications occur in up to 30% of cases, most commonly ischemic stroke, while acute coronary syndromes are reported in only 0.2% [3,4]. In some patients, abrupt hemodynamic collapse may result from either embolization or transient obstruction of the mitral orifice, the so-called “ball-valve effect” [2,3]. Echocardiography remains the cornerstone of diagnosis, with transesophageal imaging offering superior sensitivity when transthoracic views are limited [3].

During mass gatherings such as Hajj and Umrah (Islamic pilgrimages to Makkah), elderly individuals with unrecognized cardiovascular disease are particularly vulnerable to sudden collapse [5]. Differentiating ischemic heart disease from structural cardiac lesions in the pre-hospital setting is especially difficult when both neurological and cardiac manifestations coexist. We report the case of a 77-year-old woman who collapsed during *Tawaf *(circumambulation of the Kaaba in Makkah) and was subsequently found to have a left atrial myxoma presenting with the rare triad of pulseless electrical activity arrest, ST-segment elevation myocardial infarction (STEMI), and multifocal embolic strokes.

## Case presentation

A 77-year-old woman with a history of type 2 diabetes mellitus collapsed while performing *Tawaf* during Umrah. Eyewitnesses reported the sudden onset of chest pain followed by immediate loss of consciousness. On arrival, pre-hospital emergency medical services found her unresponsive and pulseless, in pulseless electrical activity (PEA). Return of spontaneous circulation (ROSC) was achieved after approximately four minutes of high-quality cardiopulmonary resuscitation (CPR).

At the nearest emergency department, the patient was intubated and deeply comatose, with a Glasgow Coma Scale (GCS) score of 3 (Eye: 1, Motor: 1, Verbal: intubated). She remained hemodynamically unstable and required vasopressor support with norepinephrine and vasopressin. Laboratory investigations showed leukocytosis (17 × 10⁹/L), anemia (hemoglobin 10 g/dL), elevated serum creatinine (190 µmol/L), and a mildly elevated troponin level (0.15 ng/mL), consistent with acute kidney injury and myocardial injury.

**Figure 1:**
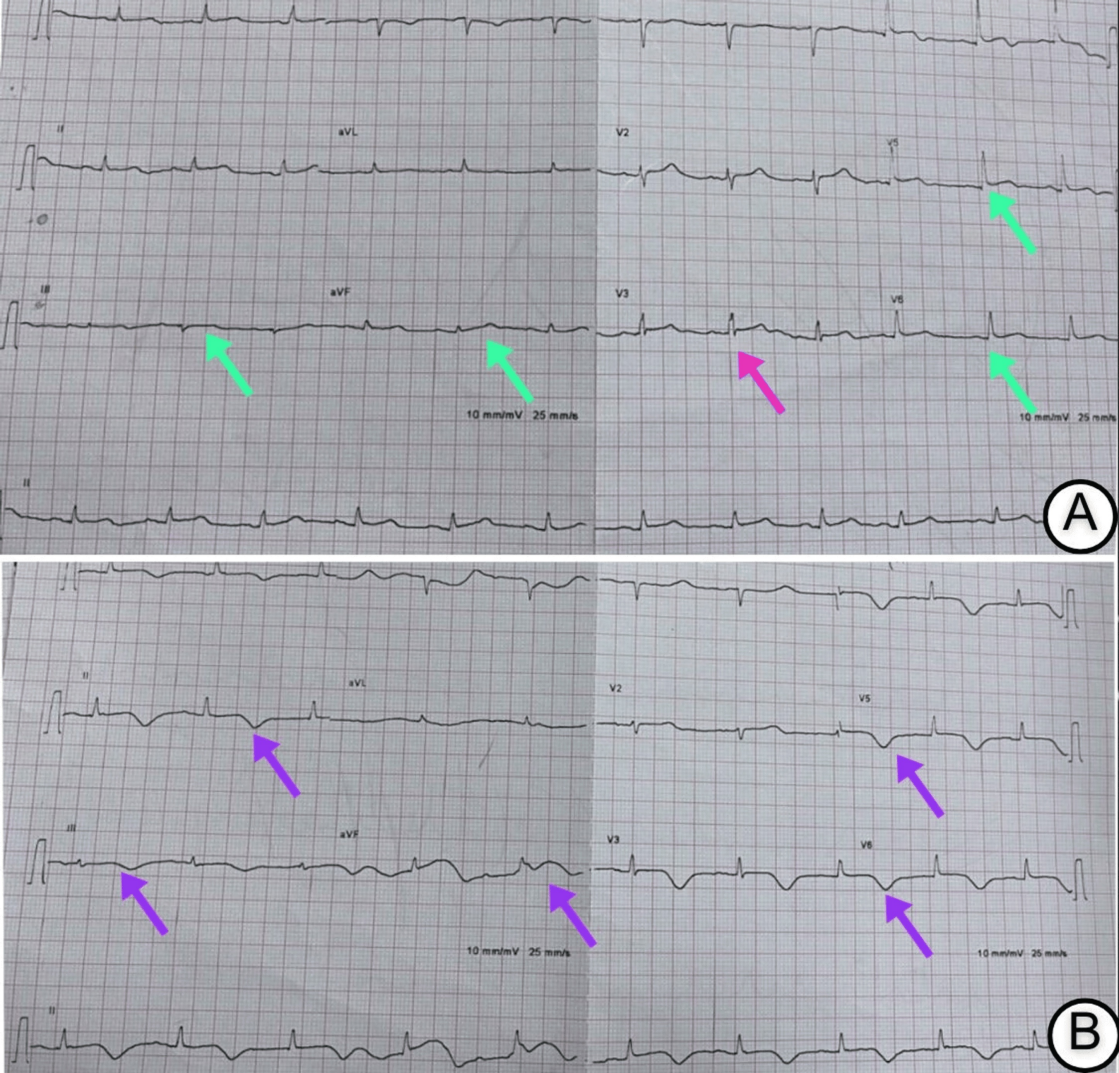
Electrocardiographic findings (A) Twelve-lead ECG obtained immediately after return of spontaneous circulation demonstrates ST-segment elevations (green arrows) in the inferior leads (II, III, aVF) and lateral leads (V5–V6), along with ST-segment depression (pink arrow) in lead V3, consistent with acute myocardial infarction. (B) Repeat ECG four hours later demonstrates deep T-wave inversions (purple arrows) in the inferior (II, III, aVF), lateral (V5–V6), and anterior (V2–V3) territories, indicative of evolving ischemia.

**Figure 2:**
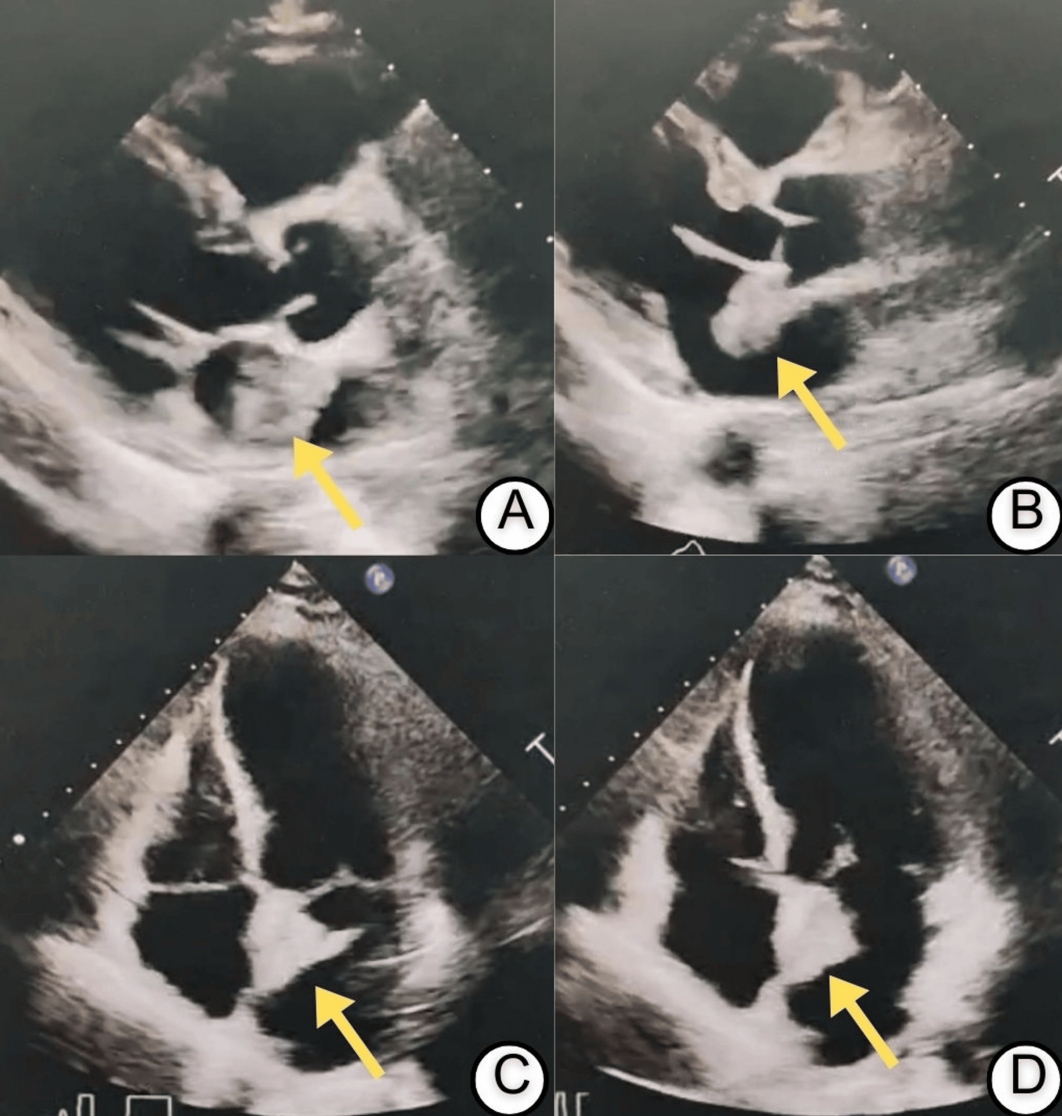
Transthoracic echocardiography demonstrating a left atrial myxoma. (A, B) Parasternal long-axis views showing a large, pedunculated, and mobile mass (yellow arrows), measuring approximately 2.6 × 3.2 cm, attached to the interatrial septum and prolapsing across the mitral valve into the left ventricle during diastole. (C, D) Apical four-chamber views confirming the left atrial mass with dynamic prolapse into the left ventricle, consistent with a left atrial myxoma.

Electrocardiography (ECG) demonstrated ST-segment elevations in the inferior and lateral leads (Figure 1A). A repeat ECG four hours later revealed deeply inverted T waves in the same territories (Figure 1B). Transthoracic echocardiography identified a large, pedunculated, mobile mass measuring 2.6 × 3.2 cm, arising from the interatrial septum and prolapsing into the left ventricle during diastole (Figure 2). Regional wall motion abnormalities were also noted in the left anterior descending (LAD) artery territory.

**Figure 3:**
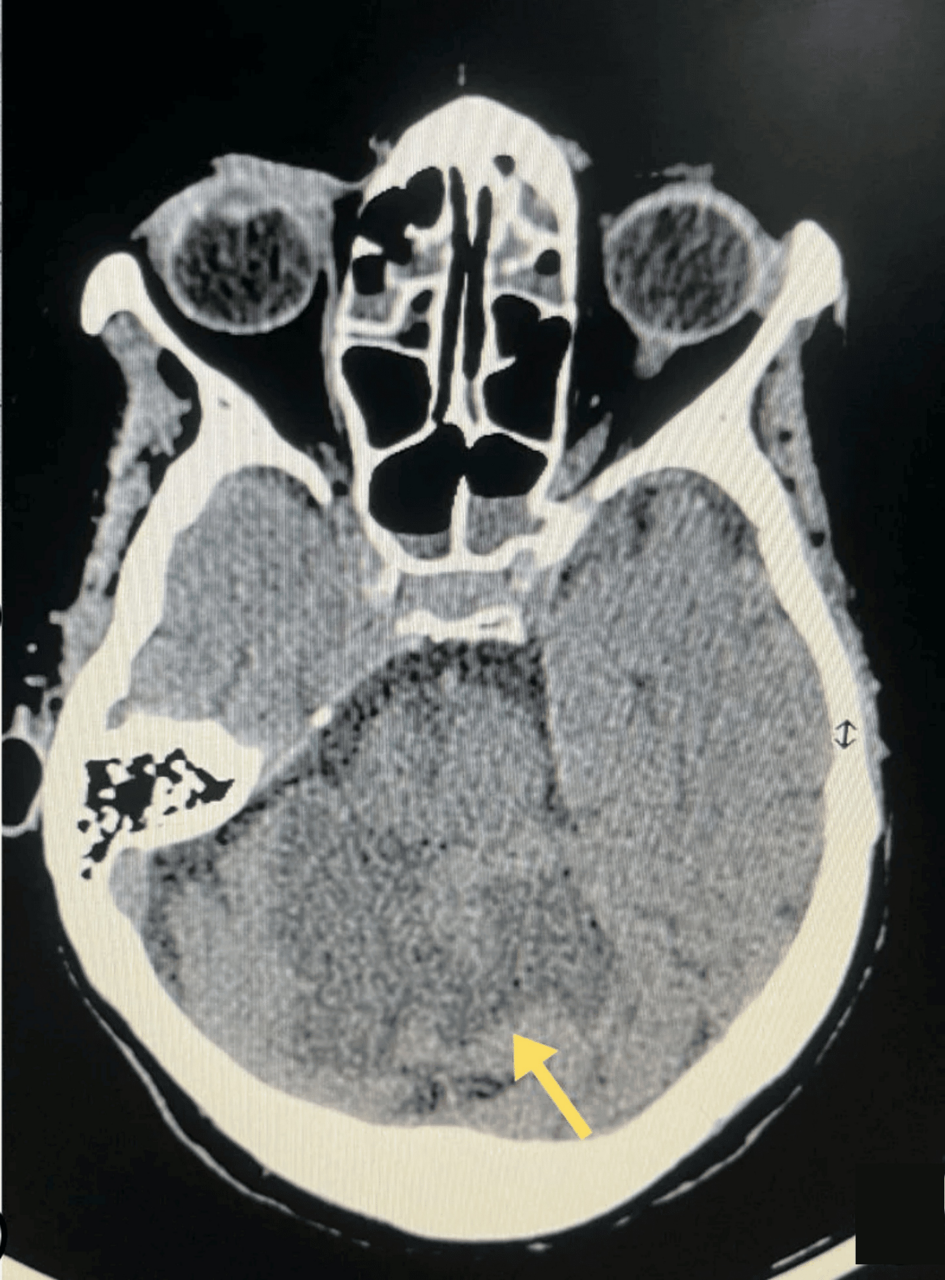
Axial non-contrast CT brain demonstrating acute cerebellar infarction (yellow arrow) without intracranial hemorrhage

After sedation was discontinued, the patient’s neurological status remained impaired. Non-contrast computed tomography (CT) of the brain demonstrated bilateral superior cerebellar infarctions, a right pontine infarction, and additional infarcts in the left inferior cerebellum and left thalamus, without evidence of hemorrhage (Figure 3). Neurological examination showed asymmetrical weakness: right upper limb power 3/5, right lower limb 4/5, left upper limb 1/5, and left lower limb 2/5.

After intracranial hemorrhage was excluded, dual antiplatelet therapy and a continuous intravenous heparin infusion were initiated for secondary prevention of embolic events. Over the subsequent three days, the patient demonstrated gradual neurological improvement, with her GCS improving to 11 (Eye: 4, Motor: 5, Verbal: 2 (non-verbal due to intubation)). She regained strength in her right-sided limbs and showed partial recovery on the left side. Following multidisciplinary evaluation and stabilization, she was transferred to a tertiary cardiac surgery center for definitive excision of the atrial mass.

## Discussion

This case demonstrates an exceptionally rare convergence of pulseless electrical activity arrest, inferior STEMI, and multifocal posterior circulation strokes, all caused by a single atrial myxoma. While embolic stroke is reported in up to 30% of patients with myxomas [3], and acute myocardial infarction has been documented in only 0.2% [4], the simultaneous occurrence following cardiac arrest remains extraordinary.

**Figure 4:**
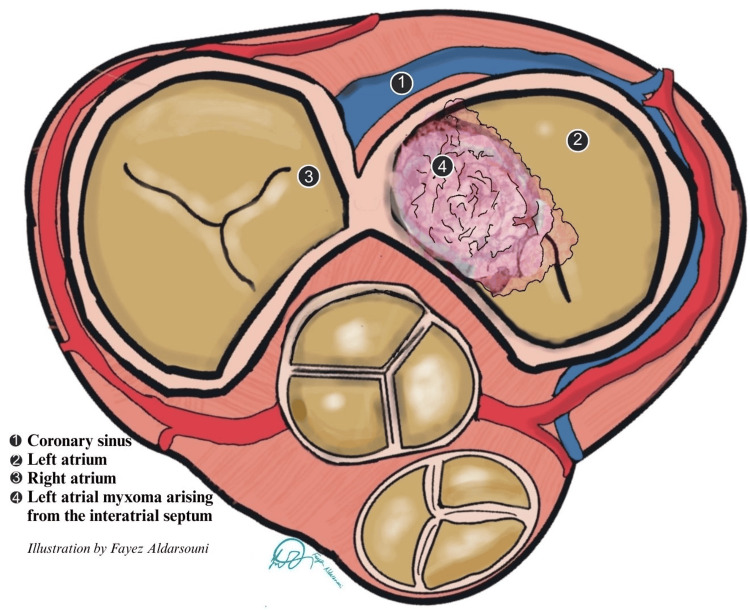
Schematic illustration of a left atrial myxoma. Cross-sectional diagram of the atria showing: (1) the coronary sinus, (2) the left atrium, (3) the right atrium, and (4) a left atrial myxoma arising from the interatrial septum. The tumor is depicted as a pedunculated, friable mass occupying the left atrial cavity. Image Credit: Fayez Aldarsouni (Author)

The tumor’s morphology was pivotal in producing this presentation. The pedunculated, highly mobile mass prolapsed across the mitral orifice during diastole (Figure 4), predisposing to intermittent inflow obstruction and acute hemodynamic collapse, a mechanism described as the “ball-valve effect” [3]. At the same time, the friable surface of the tumor provided a substrate for embolization. Coronary embolism best explains the inferior STEMI, while the widespread posterior circulation infarcts reflect tumor fragments or thrombus entering the vertebrobasilar system. Such dual vascular involvement has been documented but remains exceedingly rare [1,2].

In our patient, persistent hypotension after ROSC was likely multifactorial. Acute coronary embolization could have impaired contractility, while intermittent prolapse of the tumor across the mitral orifice may have contributed to dynamic obstruction of left ventricular inflow. In addition, the possibility of global post-arrest myocardial stunning cannot be excluded. Bedside echocardiography provided crucial information by demonstrating the atrial mass and helping to weigh these differential contributors rather than attributing instability solely to presumed myocardial dysfunction [3].

Assessment of neurological status was complicated by sedation and coma after arrest, which limited the detection of focal deficits. In such contexts, reliance on imaging becomes decisive. Engberding et al. have emphasized that transthoracic echocardiography often identifies the mass, but transesophageal echocardiography provides superior sensitivity (100% vs 95%) and is particularly valuable in intubated or critically ill patients where acoustic windows are poor [3,6]. Similarly, MRI has been reported to delineate infarct burden and to help differentiate tumor emboli from thrombotic stroke [3].

The decision to begin dual antiplatelet therapy together with anticoagulation illustrates a therapeutic dilemma. Stroke guidelines generally advise delaying anticoagulation for large infarcts to reduce the risk of hemorrhagic transformation [7]. However, with a highly mobile myxoma acting as an ongoing embolic source, and concurrent coronary embolism producing STEMI, immediate therapy was justified.

The timing of surgical excision remains controversial when stroke complicates atrial myxoma. Surgery eliminates the embolic source but requires cardiopulmonary bypass with systemic heparinization, which may precipitate hemorrhagic transformation of a recent infarct. While some reports describe delaying surgery for several weeks after major infarcts [2], others support urgent removal in smaller or stable strokes [1]. Large series have shown that operative mortality is generally low (0-3%) and long-term survival exceeds 95%, but perioperative embolization remains a recognized risk [3,8]. In this case, a short stabilization period with close neurological monitoring preceded transfer for resection, with the decision weighed against the dual risks of further embolism and intraoperative bleeding.

Taken together, this case underscores how atrial myxoma can masquerade as common cardiovascular emergencies. Due to the rarity of this differential, formal recommendations for such presentations in the context of mass gatherings remain absent; yet, awareness of structural cardiac causes and early use of echocardiography may prevent misdiagnosis and inappropriate therapies [9]. In particular, during the Hajj and Umrah season, when millions of pilgrims of diverse ethnic backgrounds and variable health profiles converge [10], the burden of both age-related disease and uncommon pathologies may be amplified [5].

## Conclusions

This case highlights how a left atrial myxoma can simultaneously cause cardiac arrest, STEMI, and multifocal stroke. Early echocardiography, cautious antithrombotic use, and timely surgery were pivotal. In mass gatherings, structural cardiac lesions must remain a differential, with point-of-care echo crucial for rapid, accurate triage.
